# Plasticity of ventral tegmental area disturbance during abstinence after repeated amphetamine exposure: restoration by selective activation of group II metabotropic glutamate receptors

**DOI:** 10.3389/fphar.2025.1534101

**Published:** 2025-04-23

**Authors:** Ornella Valenti, Katarzyna Anna Rekawek, Sophie Wieser, Hilal Bulut, Petra Scholze, Stefan Boehm

**Affiliations:** ^1^ Division of Neurophysiology and Neuropharmacology, Center for Physiology and Pharmacology, Medical University of Vienna, Vienna, Austria; ^2^ Molecular Biotechnology, Fachhochschule (FH) Campus Wien, Vienna, Austria; ^3^ Division of Pathobiology of the Nervous System, Center for Brain Research, Medical University of Vienna, Vienna, Austria

**Keywords:** amygdala, amphetamine, avoidance behavior, dopamine neurons, metabotropic glutamate receptors, nucleus accumbens, ventral tegmental area, ventral hippocampus

## Abstract

**Background and aims:**

The psychostimulant actions of amphetamine (AMPH) have been correlated with its ability to orchestrate ventral tegmental area (VTA) dopamine (DA) neuron activity states and, thus, DA release in output regions: in rats, a single exposure is sufficient to reduce the fraction of spontaneously active DA neurons, i.e., DA neuron population activity, whereas AMPH abstinence after repeated exposure leads to an increase. Here, this switch in DA neuron activity was resolved in detail in mice, and its sensitivity towards activation of group II metabotropic glutamate receptor (mGluR2 and mGluR3) was investigated.

**Experimental procedure:**

All experiments were conducted on C57BL/6J male mice. After repeated AMPH administration (2 mg/kg), the amine was withdrawn for up to 15 days and VTA DA neuron activity was assessed. The involvement VTA afferent regions with respect to AMPH actions was analyzed either by local instillation of drugs or through inactivation by tetrodotoxin. Selective agonists or allosteric modulators of mGluR2 and mGluR3 were used to explore whether group II mGluR might interfere with VTA disturbances caused by the amine.

**Results:**

After repeated AMPH exposure, VTA DA neuron activity remained reduced for 4 days and then rose to a hyperdopaminergic state within 15 days. The initial hypodopaminergia was coordinated by an amygdala (AMG) - nucleus accumbens (NAc) -VTA pathway, whereas the hyperactivity relied on ventral hippocampus (vHPC). Hypodopaminergic VTA activity was recovered towards physiological levels by activation of mGluR2, but not mGluR3, and this remission was contingent on glutamatergic transmission within NAc and propagation via the ventral pallidum. Results of a light-dark transition task confirmed anxiolytic efficaciousness of mGluR2 activation. The hyperdopaminergic VTA activity, in contrast, was normalized by selective activation of mGluR3, but not mGluR2, within vHPC. AMPH re-exposure after abstinence turned VTA activity down, but this suppression involved alternative circuits and could no longer be rescued by mGluR activation.

**Conclusion:**

Thus, abstinence from repeated AMPH intake drives VTA activity from hypo-into hyperdopaminergic states, and both can be readjusted towards physiological levels via different members of group II mGluRs.

## 1 Introduction

The sympathomimetic drug amphetamine (AMPH) is used to treat attention deficit hyperactive disorder, narcolepsy, and obesity; due to its psychostimulant effects, the amine is often consumed for recreational purposes or to ameliorate performance (Wood et al., 2014).

The ventral tegmental area (VTA) and the nucleus accumbens (NAc), core nuclei of the mesolimbic dopamine (DA) system, are affected most by recurrent AMPH use, and disruptions therein emerge during sensitization (Volkow et al., 2009; Sesack and Grace, 2010). AMPH impinges on VTA DA neurons via D1 receptors (Bjijou et al., 1996) and enhances DA release in NAc (Di Chiara and Imperato, 1988; Koob, 1992; Oliva and Wanat, 2016; Schultz, 2019). The main output of NAc are GABAergic medium spiny neurons (MSNs); these comprise two major types of neurons that either express dynorphin and DA D1 receptors (D1-MSN) or enkephalin and D2 receptors (D2-MSN) and connect NAc to VTA by a direct and indirect pathway, respectively (Gerfen, 2004). In psychostimulant sensitization, the two MSN populations appear to oppose each other: thus, reduction of D2-MSN activity facilitated AMPH sensitization, whereas disruption of the direct pathway led to the opposite effect (Ferguson et al., 2011; Pascoli et al., 2012; Bock et al., 2013; Stefanik et al., 2013; Heinsbroek et al., 2017).

Psychostimulant sensitization does not only affect MSNs as NAc output, but also afferent systems, the most relevant being prefrontal cortex (PFC), amygdala (AMG) and ventral hippocampus/subiculum (vHPC) (Pierce and Kalivas, 1997; Volkow et al., 2009; Volkow et al., 2013; Sesack and Grace, 2010; Yager et al., 2015; Koob and Volkow, 2016; Lüscher, 2016; Wearne and Cornish, 2019). A multi-nuclei pathway comprising vHPC, NAc, and ventral pallidum (VP) controls the fraction of DA neurons exhibiting spontaneous firing (i.e., *DA neuron population activity*) in rats, which determines tonic DA release in VTA output structures (Floresco et al., 2003; Lisman and Grace, 2005; Grace et al., 2007). Activation of vHPC pyramidal neurons increases firing in GABAergic MSNs and thereby causes inhibition of VP neurons; consequently, VTA DA neurons are disinhibited and disposed to fire (Floresco et al., 2003; Grace et al., 2007; Belujon and Grace, 2011). AMG regulates VTA by stimulating VP GABAergic projections that suppress VTA DA neuron activity (Belujon and Grace, 2011; Chang and Grace, 2014). Additionally, AMG can control mesolimbic DA neuron states via NAc, and release of DA, in turn, orchestrates AMG activity; moreover, a glutamatergic path from AMG to NAc appeared to control seeking behaviour (Di Ciano and Everitt, 2004; Gremel and Cunningham, 2009; Lintas et al., 2012; Xu et al., 2024). PFC governs VTA activity by regulating both, interneuron and DA neuron firing through either direct projections or via HPC and AMG (Carr and Sesack, 2000; Jackson and Moghaddam, 2001; Lodge, 2011; Patton et al., 2013; Belujon et al., 2014). Originally, acute administration of AMPH was shown to reduce population activity in rats by ∼50% (Lodge and Grace, 2008), but this finding was not confirmed in subsequent investigations (Belujon et al., 2016). Abstinence after acute AMPH exposure was found to keep population activity in rats at lowered levels, to cause AMG hyperactivity, and led to anxiety-like states (Chang and Grace, 2014; Belujon et al., 2016). By contrast, prolonged abstinence after repeated AMPH exposure elicited AMPH-sensitization (AS) and enhanced VTA DA neuron firing via vHPC projections towards VTA (Lodge and Grace, 2007; Lodge and Grace, 2008; Lodge and Grace, 2011). In mice, acute AMPH exposure reduced the fraction of spontaneously active VTA DA neurons, whereas three intermittent administrations given during conditioned place preference training elicited hyperdopaminergia (Valenti et al., 2021).

Recurrent exposure to psychostimulants induces neuroplastic alterations in molecular markers, synaptic transmission, and neuronal networks (Pierce and Kalivas, 1997; Arroyo-García et al., 2021). Underlying mechanisms involve changes in expression of ionotropic (iGluR) and metabotropic glutamate (mGluR) receptors (Chen et al., 2010). Within the mGluR family, eight different receptors (mGluR1-8) are categorized into three groups (group I to III) that differ in pharmacological characteristics, neuroanatomical distribution, and coupling to G-proteins and associated signalling cascades; mGluRs have been proposed as targets for the treatment of drug addiction and psychiatric disorders (Cleva and Olive, 2012; Duncan and Lawrence, 2012; Pomierny-Chamioło et al., 2014). With respect to AMPH, the group II mGluR (comprising mGluR2 and mGluR3) selective agonist LY379268 and the mGluR2 positive allosteric modulator (PAM) LY487379 were found to attenuate drug-induced locomotor activity to a similar extent (Galici et al., 2005). Moreover, LY379268 impeded sensitization of AMPH self-administration and locomotor activity (Kim et al., 2005), the former effect being observed with methamphetamine as well (Kufahl et al., 2013). In addition, the group II agonist LY354740 exhibited efficacy in several anxiety tests such as elevated plus maze (Monn et al., 1997; Helton et al., 1998). A recent study employed mGluR2 and mGluR3 knock-out (KO) mice to determine their role in methamphetamine sensitization: only mGluR3 KO mice exhibited methamphetamine conditioned place preference (CPP) and sensitization with respect to locomotor activity; this was taken as indication of mGluR3, but not mGluR2, activation as treatment of methamphetamine addiction (Busceti et al., 2021).

Despite all this progress, knowledge on the impact of recurrent psychostimulant exposure on cortico-limbic-VTA circuits has remained fragmentary. Understanding the impact of chronic AMPH use on the extended mesolimbic DA system is of high relevance, as the amine is not only applied in therapy, but also used illegally, and repeated intake can lead to addiction and psychotic symptoms (Berman et al., 2009; Uddin et al., 2017; Weidenauer et al., 2017). To shed more light on the reorganization of neural networks by AMPH in mice, we employed a system-oriented approach and evaluated levels of activity of DA neurons population in VTA by determination of the fraction of spontaneously active neurons (DA neuron population activity); circuit manipulation by chemicals was used to elucidate how DA neuron firing is regulated by main afferent regions during AMPH exposure and subsequent abstinence. These VTA afferents are known to govern DA neuron responses and thereby tonic DA release, and variations therein are correlated with several neuropsychiatric diseases including addiction (Grace, 2016; Solinas et al., 2019). Furthermore, we tested whether and how activation of specific subtypes of group II mGluRs might interfere with the impact of AMPH on the NAc-VTA circuit and upstream afferents. We discovered that, during the first stage of abstinence, activation of accumbal mGluR2 elevated DA neuron activity and prevented anxiety-like responses, whereas activation of mGluR3 counteracted hyperdopaminergic states associated with AS by actions in vHPC. However, all group II mGluR activators failed to reverse effects of AMPH re-exposure after drug abstinence. Thereby, these results identify selective mGluR2/3 compounds as effective tools to counteract the consequences of repeated AMPH intake and subsequent abstinence mechanisms.

## 2 Materials and methods

### 2.1 Subjects

Experiments were conducted in compliance with the ARRIVE guidelines and with the regulations of the Medical University of Vienna. All procedures were approved by the Austrian Ministry of Science (GZ 66.009/0382-WF/V/3b/2017 and 2021-0–724.206). Male adult (10–20-week-old) C57BL6/J mice were used in this study. During the early stages of our investigation, we also prerecorded DA neuron activity from the female VTA. However, we decided not to include those results as they revealed sex-dependent differences; those data are currently elaborated in a separate study focusing on sex-specific drug actions. All subjects were housed in deputed facilities with standard conditions; water and chow were provided *ab libitum* (Valenti et al., 2021). For consistency with previous rat studies (Lodge and Grace, 2008; Belujon et al., 2016; Rincón-Cortés et al., 2018) and in order to match behavioral activity with electrophysiology signatures, i.e., DA neuron population activity, all procedures were conducted between 8:30 and 9:30, during the first hours of the diurnal cycle.

### 2.2 Materials

Isoflurane (Forane) was from AbbVie (Vienna, Austria); Chicago Sky Blue from Alfa Aesar (Karlsruhe, Germany); tetrodotoxin (TTX) from Latoxan. D-AMPH sulfate, chloral hydrate, kynurenic acid and bulk chemicals were from Sigma-Aldrich (Vienna, Austria). SKF81297 ((±)-6-Chloro-2,3,4,5-tetrahydro-1-phenyl-1*H*-3-benzazepine) hydrobromide was from Hellobio; LY341495 ((2*S*)-2-Amino-2-[(1*S*,2*S*)-2-carboxycycloprop-1-yl]-3-(xanth-9-yl) propanoic acid), LY487379 (2,2,2-Trifluoro-*N*-[4-(2-methoxyphenoxy)phenyl]-*N*-(3-pyridinylmethyl)ethanesulfonamide hydrochloride) were from Tocris (Biotechne, Austria); LY2794193 ((1S,2S,4S,5R,6S)-2-Amino-4-[(3-methoxybenzoyl)amino]bicyclo[3.1.0]hexane-2,6-dicarboxylic acid) from Medchemexpress (Austria).

### 2.3 Drug protocols

**FIGURE 1:**
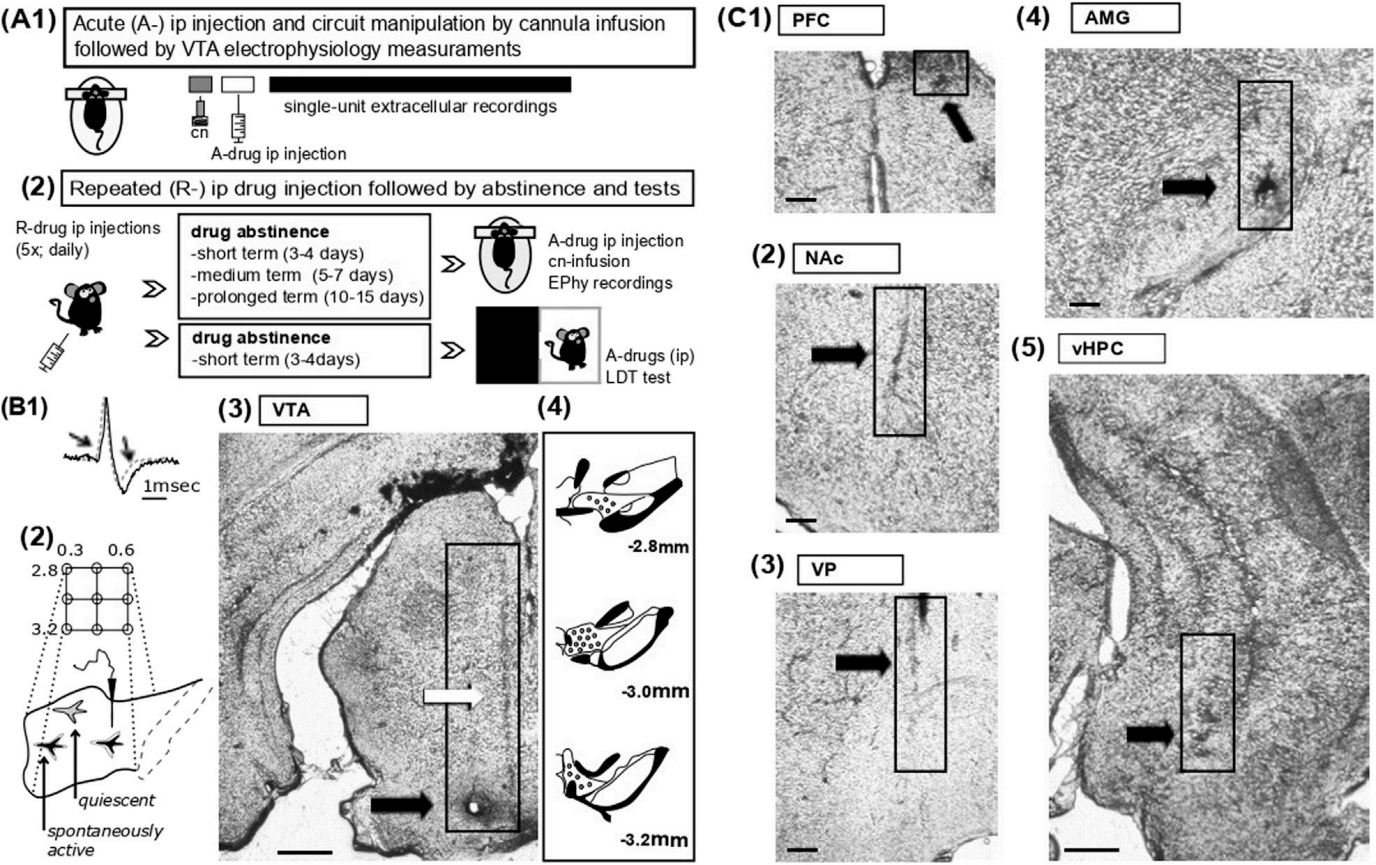
Experimental protocols for drug administration, electrophysiology and histological analysis **(A)** 1- Schematic representation of acute drug experiments. The acute effects of vehicle or AMPH on VTA DA neuron activity states were assessed under anaesthesia by single-unit extracellular recordings from the VTA; the amine was injected intraperitoneally (i.p. 2 mg/kg) prior to electrophysiology measurements. In some experiments, as indicated in the main text, specific compounds were infused into pre-selected regions via acutely implanted cannulas (cn). 2- For repeated drug experiments, vehicle or AMPH were administered repeatedly (R-drug) via the intraperitoneal route daily for five consecutive days; mice were tested at different protocols of abstinence (see methods), namely, during short-term (2–3 days from last injection), medium-term (5–6 days) and prolonged (10–15 days) abstinence. On test days, activity states of VTA DA neurons were evaluated by electrophysiological recordings in association with acute drug injections or circuit manipulations, as in A1 (see methods). The light-dark transition task (LDT) task was employed in a group of mice during short-term abstinence and following administration of acute vehicle or PAM2. **(B)** 1- Overlapping waveforms of two recorded VTA neurons, i.e., one neuron classified as ‘dopaminergic’ (black, solid line) and of one glutamatergic cell (grey, dotted line); note that the DA neuron exhibits a waveform of long-duration (>2.2 msec) characterized by prominent notch and after-hyperpolarization, both indicated by arrows. 2. Sampling of the VTA was achieved by subsequent electrode penetrations (tracks) proceeding along a grid pattern (anterior, 2.8 – posterior, 3.2; medial, 0.3 – lateral, 0.5). In physiological states, about half of the DA neuron population in VTA is typically quiescent due to hyperpolarization imposed by VP GABAergic projection neurons. 3- Post-hoc histological analysis aided to confirm the correct placements of glass electrodes. Representative coronal VTA section shows traces (indicated by white arrow) of pipette penetrations as well as a lesion (black arrow) with staining by iontophoretic application of Chicago Sky Blue at termination of the experiments (scale bars reflect 500 µm). 4- Drawing of VTA sections from anterior to posterior indicates representative sites of recordings (black dots). **(C)** Details of representative coronal sections display tissue damage (indicated by arrow and boxes) due to cannula inserts for *in-situ* drug deliveries into the PFC (1; scale bars reflect 100 µm), NAc (2; scale bars reflect 100 µm), the VP (3; scale bars reflect 100 µm), the AMG (4; scale bars reflect 100 µm), and the vHPC (5; scale bars reflect 500 µm).

When administered systemically, all drugs were dissolved in 10% Tween (10 g Tween in 100 mL PBS; vehicle, VHC) and injected via the intraperitoneal route (i.p.). For acute experiments, AMPH (2 mg/kg) was administered 30 min prior to electrophysiology recordings (see below; Figure 1A1); the effects of the amine were compared to controls injected with vehicle. The dose of AMPH was selected for consistency with previous studies in rodents (Lodge and Grace, 2008; Belujon et al., 2016) and humans (Berman et al., 2009). To assess the capability of group II mGluRs of counteracting abstinence, we employed the following compounds: a general group II mGluR agonist LY354740 (3 mg/kg) (Schoepp et al., 2003), the selective mGluR2 PAM LY487379 (30 mg/kg) (Nikiforuk et al., 2010; Mango et al., 2019), and selective mGluR3 agonist LY2794193 (10 mg/kg) (Monn et al., 2018).

Circuit dissections were conducted in anesthetized mice during electrophysiology recordings by infusion of chemicals into pre-selected regions (for cannula positions see 3.4. below and Figures 1A, C). All drugs were delivered *in-situ* via acutely implanted cannulas (guide 24GA; PlasticOne, Germany) at a rate of 0.5 μL/min and a volume of 0.3–0.8 µL (Valenti and Grace, 2010; Valenti et al., 2021) (Figure 1A; see below for stereotaxic coordinates). Compounds and concentrations being instilled were the following: AMPH (10 µM) and the selective DA D1 agonist, SKF81297 (10 µM) (Maguire et al., 2014); the Na^+^ channel blocker TTX (1 µM); the ionotropic glutamate receptor antagonist kynurenic acid (5 µM) (Floresco et al., 2001), the mGluR2 PAM LY487379 (30 µM) (Mango et al., 2019), the mGluR3 selective agonist LY2794193 (10 µM) (Monn et al., 2018). As control, vehicle (DMSO, diluted 1:1,000) was delivered at a corresponding rate and volume.

For repeated AMPH administration and behavioral experiments, mice were randomly assigned to one of the protocols and single-housed at 5–7 days prior to injections. AMPH (2 mg/kg) or vehicle (10% tween) were provided by daily intraperitoneal injection for five consecutive days (Lodge and Grace, 2008). Tests occurred during drug-off periods on a pre-defined schedule; three time periods of abstinence were examined: i) 3–4 days (*short-term abstinence*), ii) 5–7 days (*medium-term abstinence*) and iii) 11–15 days (*prolonged abstinence*) from the last AMPH injection. Following drug protocols, the effects of the amine were examined by either electrophysiology recordings from VTA (see above) or behavioral tests (see below; Figure 1A2).

### 2.4 Craniotomies, electrophysiology measurements, and histology

VTA DA neuron activity states were assessed by *in-vivo* single-unit extracellular recordings under anesthesia (Figure 1); all experiments were conducted as previously described (Valenti and Grace, 2010; Valenti et al., 2021). Craniotomies exposed brain surfaces above the recording region, VTA (in mm from bregma: P: −2.8 to −3.2, L: 0.3 to 0.5, V: 4–5) (Figure 1B), and the sites of drug infusions, namely,: AMG (P: −1.5, L: 2.7, V: 3.7), NAc (A: +1.6, M: 0.7, V: 3.5), PFC (A: +1.8, M: 0.4, V: 1.3), vHPC (P: −3.6, M: 2.6, V: 3.5) and VP (P: 0, L: 1.6, V: 3.8) (Figure 1C). For all regions targeted, coordinates were calculated based on the mouse atlas (Franklin and Paxinos, 2008).

Following anesthesia with isoflurane (3% in air) and Chloral Hydrate (400 mg/kg), mice were secured on a stereotactic frame (Kopf; Germany); anesthesia was maintained with Chloral Hydrate (53 mg/kg) as needed. Microelectrodes were fabricated from borosilicate glass capillaries (1.2 mm outer diameter; Harvard Apparatus) by a vertical puller (Narishige, Japan) and filled with 0.5 M NaCl in 2% Chicago Sky Blue (*in-situ* impedance 10–25 MΩ). Pipettes were inserted within the VTA and slowly advanced from dorsal to ventral by a vertical manipulator (Scientifica, United Kingdom) for cell-hunting. The firing of spontaneously active neurons encountered during electrode penetration (track) was measured for up to 5-min. To allow sampling of neuron activity across all VTA, and assess DA neuron population activity (see below; Figures 1B2–4) at the end of the first track the electrode was retracted and moved in accordance to a predetermine path along the medio-lateral and the anterio-posterior axes (Figure 1B2); typically, five to nine vertical tracks were conducted per mouse. All signals were recorded by Spike2 software (Cambridge Electronic Design Ltd., United Kingdom) and stored for post-hoc analysis (Valenti et al., 2021).

**FIGURE 2:**
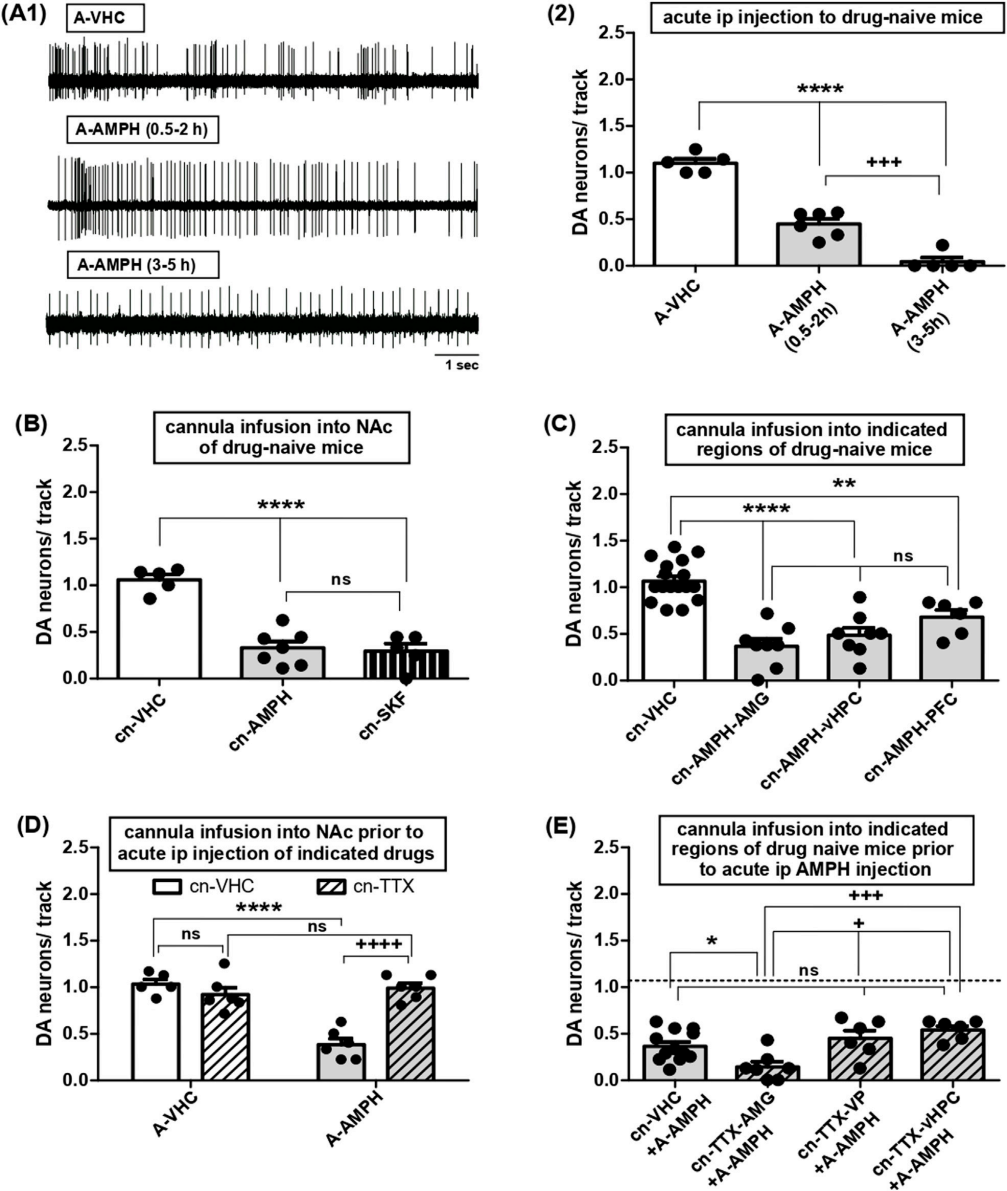
Effects of acute AMPH administration on VTA DA neuron activity and roles of various afferent circuits. **(A)** 1- Representative traces of single-unit extracellular recordings of DA neuron firing in mice injected with acute vehicle (top, A-VHC; 10% Tween) or AMPH (middle, A-AMPH; 2 mg/kg) and tested within the two time periods, i.e., between 0.5 and 2-h (middle) or 3- and 5-h (bottom) following drug administration. 2- Impact of systemic administration of VHC or AMPH on VTA DA neuron activity, i.e., the number of spontaneously firing DA neurons encountered during sampling of the VTA by consecutive electrode tracks (DA neurons/track, Y-axis) in anaesthetised mice. Results were obtained after acute i.p. injection of AMPH within 0.5- to 2-h (A-AMPH(0.5–2 h): N = 6, n = 22) or 3- to 5-h (A-AMPH(3–5 h): N = 5, n = 3) or after vehicle injection (A-VHC: N = 5, n = 43); **** indicates P < 0.0001 vs. A-VHC, +++ indicates P < 0.001 vs. A-AMPH(0.5–2 h) (One-Way ANOVA followed by Bonferroni multiple comparison; df(2,15) = 105.7, P < 0.0001). **(B)** Impact of direct infusion of VHC (cn-VHC: N = 5, n = 38), AMPH (10 μM; cn-AMPH: N = 7, n = 19), or of the DA D1 receptor agonist SKF81297 (10 μM; cn-SKF: N = 5, n = 13) via acutely implanted cannulas into the NAc on VTA DA neuron activity; ****P < 0.0001 vs. cn-VHC, ns P > 0.05 vs. cn-AMPH-vHPC (one-way ANOVA followed by Bonferroni multiple comparison; df (2,16) = 33.62, P < 0.0001). **(C)** Impact of direct infusion of AMPH via acutely implanted cannulas into AMG (cn-AMPH-AMG: N = 8, n = 23), vHPC (cn-AMPH-vHPC: N = 8, n = 31), PFC (cn-AMPH-PFC: N = 6, n = 24), or infusion of VHC (cn-VHC, all sites of infusion combined: N = 17, n = 126) on VTA DA neuron activity; ****P < 0.0001 and **P < 0.01 vs. cn-VHC; ns P > 0.05 vs. cn-AMPH-AMG (one-way ANOVA followed by Bonferroni multiple comparison; df (3,38) = 25.17, P < 0.0001). **(D)** Impact of direct infusion of VHC (cn-VHC) or TTX (1 μM; cn-TTX) into NAc of mice acutely injected intraperitoneally with vehicle (A-VHC; cn-VHC: N = 5, n = 39; cn-TTX: N = 6, n = 42) or AMPH (A-AMPH; cn-VHC: N = 6, n = 13; cn-TTX: N = 6, n = 46) on VTA DA neuron activity; ****P < 0.0001, ++++ P < 0.0001, ns P > 0.05 (two-way ANOVA followed by Bonferroni multiple comparison; treatment: df (1,19) = 21.13, P = 0.0002; circuit manipulation: df (1,19) = 15.27, P = 0.0009, interaction: df (1,19) = 32.20, P < 0.0001). **(E)** Impact of direct infusion of VHC (cn-VHC: N = 12, n = 37; all locations combined) or TTX (1 μM; cn-TTX) into AMG (cn-TTX-AMG: N = 7, n = 8), VP (cn-TTX-VP: N = 6, n = 20) or vHPC (cn-TTX-vHPC: N = 6, n = 27) of mice acutely injected intraperitoneally with AMPH (A-AMPH) on VTA DA neuron activity; *P < 0.05 vs. cn-VHC, +++ P < 0.001 and + P < 0.05 vs. cn-TTX-AMG; ns P > 0.05 vs. cn-VHC (one-way ANOVA followed by Bonferroni multiple comparison; df (3,30) = 7.383, P = 0.0009).

**FIGURE 3:**
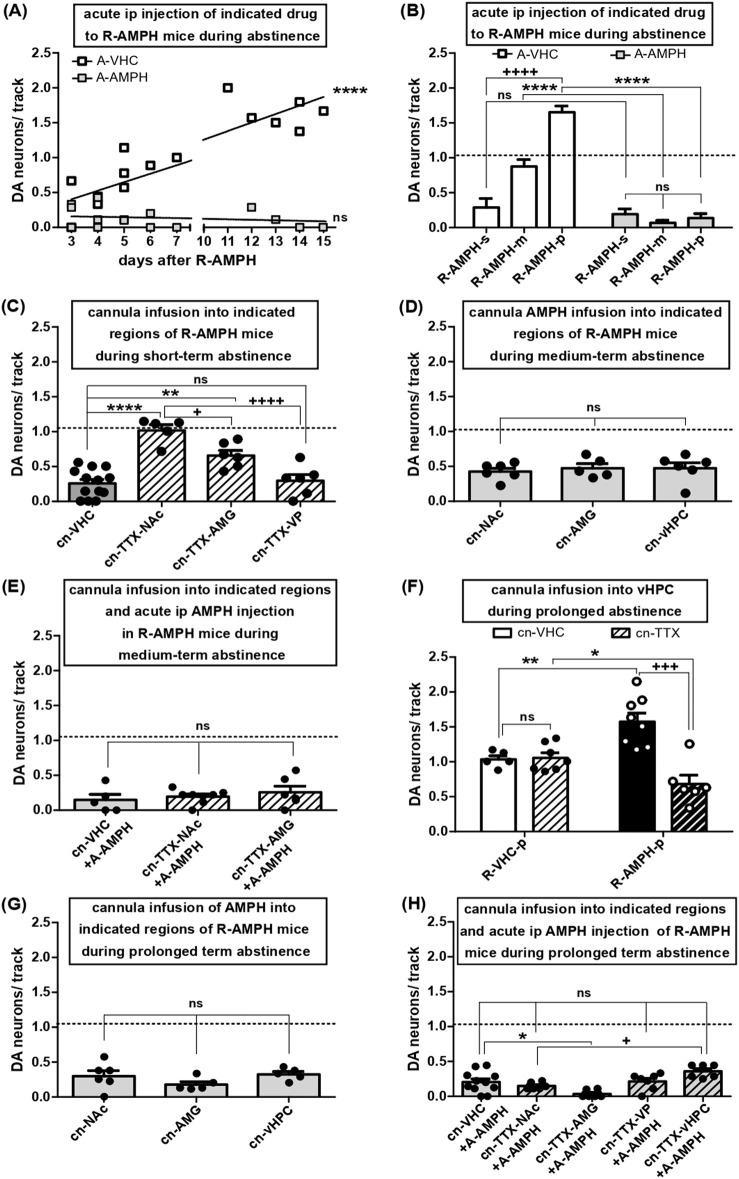
Shift in VTA DA neuron firing during abstinence after repeated AMPH exposure and effects of re-exposure: roles of various afferent circuits. **(A)** VTA DA neuron activity in anaesthetised mice after five consecutive days of AMPH exposure and subsequent abstinence for the number of days indicated on the x-axis. The results were obtained either after i.p. injection of VHC (open symbols) or after i.p. re-administration of AMPH (2 mg/kg; grey filled symbols). Linear regression of open symbol data (slope: 0.1211 ± 0.01765; R square = 0.7708; df(1, 16) = 47.09; P < 0.0001) **(B)** Same data as in A, but shown as arithmetic means +SEM and categorized according to periods of abstinence: short-term (3–4 days; R-AMPH-s), medium-term (5–7 days; R-AMPH-m), and prolonged (11–15 days; R-AMPH-p). Results obtained with VHC are shown as open bars (R-AMPH-s + A-VHC: N = 5, n = 12; R-AMPH-m + A-VHC: N = 5, n = 34; R-AMPH-p + A-VHC: N = 6, n = 67), those with AMPH as bars filled in grey (R-AMPH-s + A-AMPH: N = 6, n = 69; R-AMPH-m + A-AMPH: N = 6, n = 3; R-AMPH-p + A-AMPH: N = 5, n = 5) ****P < 0.0001, ns P > 0.05 A-AMPH vs. A-VHC; ++++ P < 0.0001 within A-VHC, different time of abstinence (two-way ANOVA followed by Bonferroni multiple comparison; treatment: df (1,27) = 136.0, P < 0.0001; time of abstinence: df (2,27) = 30.61, P < 0.0001, interaction: df (2,27) = 35.14, P < 0.0001). **(C)** Impact of direct infusion of vehicle (cn-VHC; all locations combined; N = 13, n = 29) or TTX (1 μM; cn-TTX) via acutely implanted cannulas into NAc (cn-TTX-NAc: N = 5, n = 39), AMG (cn-TTX-AMG: N = 6, n = 28), or VP (cn-TTX-VP: N = 6, n = 15) of mice during short-term abstinence from repeated AMPH exposure on VTA DA neuron firing. ****P < 0.0001, **P < 0.01, ns P > 0.05 vs. VHC; ++++ P < 0.0001, + P < 0.05 vs. cn-TTX-NAc (one-way ANOVA followed by Bonferroni’s multiple comparison; df (3,29) = 21.01, P < 0.0001). **(D)** Impact of direct infusion of AMPH (10 µM) via acutely implanted cannulas into either NAc (cn- NAc: N = 6, n = 21), AMG (cn -AMG: N = 5, n = 16), or vHPC (cn-vHPC: N = 6, n = 24) on VTA DA neuron activity during medium-term abstinence after repeated AMPH exposure; ns P > 0.05 vs. cn-NAc (one-way ANOVA followed by Bonferroni multiple comparison; P = 0.8233). **(E)** Impact of direct infusion of VHC (cn-VHC; all locations combined; N = 5, n = 5) or TTX (1 μM; cn-TTX) via acutely implanted cannulas into NAc (cn-TTX-NAc: N = 8, n = 14) or AMG (cn-TTX-AMG: N = 6, n = 12) of mice during medium term abstinence from repeated AMPH exposure and injected i.p. acutely with AMPH (2 mg/kg; A-AMPH) on VTA DA neuron activity. ns P > 0.05 vs. cn-VHC (one-way ANOVA followed by Bonferroni multiple comparison; P = 0.5376). **(F)** Impact of direct infusion of VHC (cn-VHC) or TTX (1 μM; cn-TTX) into vHPC of mice during prolonged abstinence after either repeated vehicle (R-VHC-p; cn-VHC: N = 5, n = 38; cn-TTX: N = 7, n = 56) or AMPH (R-AMPH-p; cn-VHC: N = 7, n = 92; cn-TTX: N = 6, n = 33) exposure on VTA DA neuron activity. +++ P < 0.001, **P < 0.01, *P < 0.05 (two-way ANOVA followed by Bonferroni multiple comparison; treatment: P = 0.4451; circuit manipulation: F(1,22) = 26.61, P = 0.0006, interaction: df (1,22) = 29.42, P = 0.0003). **(G)** Impact of direct infusion of AMPH (10 µM) via acutely implanted cannulas into either NAc (cn- NAc: N = 6, n = 14), AMG (cn-AMG: N = 5, n = 7) or vHPC (cn-vHPC: N = 5, n = 12) of mice during prolonged abstinence after repeated exposure on VTA DA neuron population activity (one-way ANOVA followed by Bonferroni multiple comparison; P = 0.2383). **(H)** Impact of direct infusion of VHC (cn-VHC; all locations combined; N = 11, n = 19) or TTX (1 μM; cn-TTX) via acutely implanted cannulas into either NAc (cn-TTX-NAc: N = 6, n = 6), AMG (cn-TTX-AMG: N = 6, n = 2), VP (cn-TTX-VP: N = 7, n = 12) or vHPC (cn-TTX-vHPC: N = 6, n = 18) of mice during prolonged abstinence from repeated AMPH exposure and injected i.p. acutely with AMPH (2 mg/kg; A-AMPH) on VTA DA neuron activity; *P < 0.05, ns p > 0.05 vs. cn-VHC; + P < 0.05 vs. cn-TTX-NAc (one-way ANOVA followed by Bonferroni multiple comparison; df (4,35) = 6.736, P = 0.0005).

**FIGURE 4:**
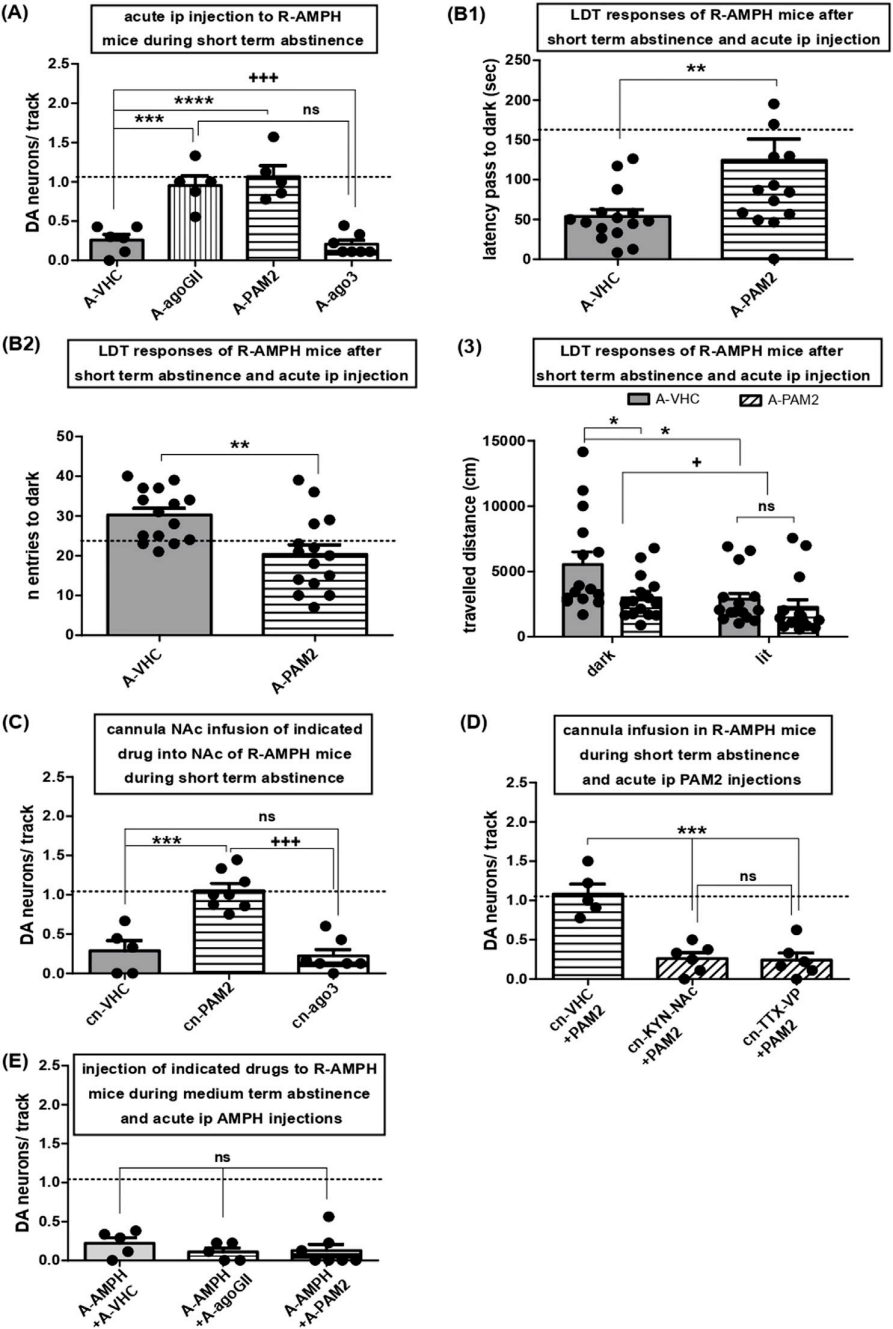
Activation of mGluR2, but not mGluR3, rescues hypodopaminergic VTA activity after short-term abstinence and attenuates anxiety-like behavior: role of the indirect NAc VTA connection and lack of effect on AMPH re-exposure. **(A)** Impact of acute i.p. injections of either VHC (A-VHC: N = 6, n = 12) or the general group II mGluR agonist LY354740 (3 mg/kg; A-agoGII: N = 5, n = 43), the selective mGluR2 PAM LY487379 (30 mg/kg; A-PAM2: N = 5, n = 43), and the selective mGluR3 agonist LY2794193 (10 mg/kg; A-ago3: N = 7, n = 24) on VTA DA neuron population activity in mice during short-term abstinence after repeated AMPH exposure; ****P < 0.0001, ***P < 0.001, ns P > 0.05 vs. A-VHC; +++ P < 0.001 vs. A-agoGII (one-way ANOVA followed by Bonferroni multiple comparison; df (3,22) = 22.86, P < 0.0001). **(B)** Following repeated AMPH exposure and short-term abstinence, mice were challenged by a LDT task after i.p. injections of either vehicle (A-VHC: N = 15) or the mGluR2 PAM (A-PAM2: N = 15). 1- Shows the latency to the first escape from the bright chamber into the dark (first latency) **P < 0.01 vs. A-VHC (Unpaired t-test; t(28) = 2.509, P = 0.0091). 2- Displays the total number of entries into the dark chamber. **P < 0.01 vs. A-VHC (Unpaired t-test; t(28) = 3.350, P = 0.0012). 3- Shows the distance travelled during navigation of the dark or lit compartment for mice injected with repeated AMPH and, following short-term abstinence, injected with either VHC or mGluR2 PAM, *P < 0.05, + P < 0.05 (two-way ANOVA followed by Bonferroni multiple comparison; interaction, P = 0.140; treatment, df (1,56) = 5.91, P = 0.0183; compartment, df (1,56) = 6.63, P = 0.0127). **(C)** Impact of direct infusion of VHC (cn-VHC: N = 5, n = 12), the mGluR2 PAM LY487379 (30µM; cn-PAM2: N = 8, n = 61), or the selective mGluR3 agonist LY2794193 (10µM; cn-ago3: N = 7, n = 10) via acutely implanted cannulas into the NAc of mice during short-term abstinence from repeated AMPH exposure on VTA DA neuron activity. ***P < 0.001 vs. cn-VHC, ns P > 0.05 vs. cn-VHC, +++ P < 0.0001 vs. cn-PAM2 (one-way ANOVA followed by Bonferroni multiple Comparison Test; df (2,19) = 25.99, P < 0.0001). **(D)** Impact of direct infusion of VHC (cn-VHC: N = 5, n = 47) and kynurenic acid (30µM; cn-KYN-NAc: N = 6, n = 13), respectively, via acutely implanted cannulas into NAc or of TTX into VP (cn-TTX-VP: N = 6, n = 12) prior to i.p. mGluR2 PAM (+PAM2; LY487379) injection on VTA DA activity in mice during short-term abstinence from repeated AMPH exposure. ***P < 0.001 vs. cn-VHC, ns P > 0.05 vs. KYN-NAc (one-way ANOVA followed by Bonferroni multiple comparison; df (2,16) = 23.41; P < 0.0001). **(E)** Impact of i.p. injection of VHC (A-VHC: N = 5, n = 12), the general group II mGluR agonist LY354740 (3 mg/kg; A-agoGII: N = 6, n = 5), or the mGluR2 PAM LY487379 (30 mg/kg; A-PAM2: N = 7, n = 8) in mice during prolonged abstinence from repeated AMPH exposure and injected i.p. with AMPH (2 mg/kg) on VTA DA neuron activity; ns P > 0.05 vs. A-VHC (one-way ANOVA followed by Bonferroni multiple comparison; P = 0.5544). Dashed lines indicate baseline DA neuron population activity.

At the end of the experiments, pipette positions were marked by electrophoretic ejection of Chicago Sky Blue (Fintronics Inc.; United States). Histological analysis was conducted to confirm the location of both, microelectrodes and cannulas (Valenti et al., 2021) (Figures 1B, C).

### 2.5 Light-dark transition task

Light-dark transition task (LDT) test was performed in a separate room by established protocols (Dorninger et al., 2019). We employed a commercially available apparatus consisting of aligned chambers plus insert (MedAssociate; United States); each insert containing two connected compartments, one dark (0 lux) and the second brightly illuminated (300 lux). Mice were repeatedly injected with either vehicle or AMPH (see above) (Figure 1A2). During short-term abstinence, subjects were transported to a deputed behavioral room 60-min before test and administered via i.p. injection with either LY487379 (30 mg/kg) or vehicle. At start, mice were positioned on the illuminated (lit) compartment of the chamber; this allowed to determine anxiety states as the tendency to take actions in order to avoid unpleasant situations (avoidance behavior). Exploration of the two compartments was recorded for 15 min. We calculated: (1) the first latency to access the dark compartment, (2) the number of entries into the dark chamber and (3) locomotor activity within the two compartments, dark and lit. All sessions were recorded using the Active Monitor Software (MedAssociate; United States).

### 2.6 Data analysis and statistics

Prior to the assessment of DA neuron population activity and behavioral responses, sample size was determined by power analysis and implemented by previous experience; however, given the low number of DA neurons recorded following acute or short-term abstinence, the size of the sample was augmented for some groups in the attempt to reach statistical significance of DA neuron activity states (i.e., firing rates and patterns; see below).

For electrophysiology recordings, spikes were extracted by employing principal component analysis of the waveforms and built-in scripts of Spike2 software; VTA cells were identified post-hoc as DA neurons based on well-established criteria (Grace and Bunney, 1984a; 1984b; Ungless and Grace, 2012; Valenti et al., 2021) (Figure 1B1). For each given mouse, VTA DA neuron population activity was determined by counting the number of recorded DA neurons (see above) and dividing the obtained value by the number of electrode tracks (DA neurons/track) performed during electrophysiology recordings (Figure 1B2). The analysis of firing rates and burst activity was also performed as reported elsewhere (Grace and Bunney, 1984a; 1984b; Lodge and Grace, 2008; Valenti and Grace, 2010; Valenti et al., 2021). However, due to the low number of neurons recorded during short-term abstinence, statistical significance could not be reached for firing rates and burst activity (burst firing was defined as the occurrence of two spikes with an interspike interval, ISI, <80 m indicating the initiation of a burst, and with two spikes occurring at an ISI >160 m pinpointing burst termination (Grace and Bunney, 1984b; Valenti and Grace, 2010; Valenti et al., 2021); therefore, we decided to show only results on VTA DA neuron population activity.

Statistical analyses were carried out by GraphPad; a general level of probability (P) <0.05 was assumed as significant. Following tests to assess normal data distribution, two groups were compared by student not paired t-test, multiple groups by one-way ANOVA followed by Bonferroni’s multiple comparison.

All data are indicated as means ± SEM. For electrophysiology, N indicates numbers of VTA recordings, n gives numbers of neurons; for behavioral studies, N indicates numbers of mice.

## 3 Results

### 3.1 Acute AMPH administration reduces tonic DA neuron activity via multiple afferents converging onto NAc

In drug naïve animals, AMPH had been found to halve the proportion of spontaneously active VTA DA neurons upon systemic administration (Lodge and Grace, 2008; Valenti et al., 2021), but the respective kinetics have remained unexplored. To evaluate onset and duration, VTA recordings were performed within 2-h or from 3- to 5-h after i.p. application. Within the first 2-h, DA neuron activity was reduced by about 50% and reached values close to zero 3 to 5 hours after administration (Figures 2A1–2; one-way ANOVA followed by Bonferroni multiple comparison; df(2,15) = 105.7, P < 0.0001).

The ‘*DA motive system’* that comprises the VTA neurons recorded above is coordinated by afferent brain areas such as vHPC, AMG, and PFC, that converge onto NAc (Volkow et al., 2017). To confirm the central role of NAc, AMPH was applied directly into that nucleus, and the fraction of active DA neurons became reduced as after systemic administration (Figure 2B; one-way ANOVA followed by Bonferroni multiple comparison; df(2,16) = 33.62, P < 0.0001) and reported before (Valenti et al., 2021). In those experiments, AMPH was shown to bypass VP (Valenti et al., 2021) thus indicating that the direct D1 receptor dependent pathway was involved. This was corroborated here, as activation of NAc D1-MSN by local infusion of SKF81297 reduced tonic DA neuron activity to the same extent as AMPH (Figure 2B). To explore whether it can orchestrate VTA via other nuclei, AMPH was delivered into AMG, vHPC and PFC, respectively, and the resulting decrease in DA neuron activity was always the same (Figure 2C; one-way ANOVA followed by Bonferroni multiple comparison; df(3,38) = 25.17, P < 0.0001). To evaluate whether these areas might be required for systemic AMPH effects, TTX was deposited locally prior to i.p. application of the amine: instillation of TTX into NAc prevented effects of AMPH (Figure 2D; two-way ANOVA followed by Bonferroni multiple comparison; treatment: df(1,19) = 21.13, P = 0.0002; circuit manipulation: df(1,19) = 15.27, P = 0.0009, interaction: df(1,19) = 32.20, P < 0.0001), but inactivation of VP or vHPC failed to do so (Figure 2E), whereas AMG blockage by TTX lowered DA neuron responses even further (Figure 2E; one-way ANOVA followed by Bonferroni multiple comparison; df(3,30) = 7.383, P = 0.0009). As TTX deposits in the PFC are known to lower VTA DA neuron firing even in the absence of AMPH (Patton et al., 2013), this area was not tested further. Introduction of TTX into VP did not alter inhibitory actions of systemic AMPH (Figure 2E) thereby confirming that the indirect pathway was not involved. Taken together, during one first exposure AMPH can impinge on tonic VTA DA neuron firing via various afferent regions including vHPC, AMG, and PFC. Nevertheless, these areas are dispensable for the effects of AMPH on VTA DA neuron activity to occur, while the latter rely on NAc and the direct NAc-VTA pathway.

### 3.2 During abstinence after repeated AMPH exposure, VTA activity slowly switches from hypo-to hyperdopaminergic states as dictated by different afferents

VTA DA neuron activity remains diminished for up to 48-h after one first AMPH exposure (Belujon et al., 2016), but equivalent effects have not been reported after repeated drug exposure. Therefore, i.p. AMPH was given on five consecutive days, and DA neuron activity states were assessed three to 15 days later (Figures 3A, B). Using this pattern of drug exposure and abstinence, mice injected with vehicle displayed 1.09 ± 0.05 (N = 16, n = 131) spontaneously active DA neurons per track, which is consistent with previous results in rats and mice and represent the physiological levels of DA neuron population activity in VTA (Lodge and Grace, 2008; Valenti et al., 2011a; 2021). After repeated AMPH exposure, DA neuron count remained reduced for three to four days of abstinence, but then rose in a linear manner to yield values of about 1.5 after 11–15 days (Figure 3A; linear regression of open symbol data: slope = 0.1211 ± 0.01765; R square = 0.7708; df(1, 16) = 47.09; P < 0.0001). Re-exposure to systemic AMPH after any abstinence brought VTA DA neuron activity to values between zero and 0.5 (Figures 3A, B). Thus, subsequent to recurrent AMPH application, DA neuron activity remained depressed for three to four days, slowly recovered towards baseline after five to seven days, and finally turned into a hyperdopaminergic state after 11–15 days. Subsequent to each of these abstinence periods, re-administration returned the activity back to hypodopaminergic levels (Figures 3A, B; two-way ANOVA followed by Bonferroni multiple comparison; treatment: df(1,27) = 136.0, P < 0.0001; time of abstinence: df(2,27) = 30.61, P < 0.0001, interaction: df(2,27) = 35.14, P < 0.0001).

The low tonic VTA DA neuron activity associated with short-term abstinence was rescued to baseline by inactivation of NAc and partially recovered by blockage of AMG; VP-inactivation failed to cause any change (Figure 3C; one-way ANOVA followed by Bonferroni’s multiple comparison; df(3,29) = 21.01, P < 0.0001). Accordingly, depression of DA neuron firing by short-term abstinence appears to involve AMG and NAc, but not the indirect NAc-VTA pathway.

After five to six days of abstinence, DA neuron activity was at baseline and reduced by re-application of AMPH (Figures 3A, B). The decline in the number of active DA neurons was the same when the amine was deposited locally into NAc, AMG, and vHPC (Figure 3D; one-way ANOVA followed by Bonferroni multiple comparison; P = 0.8233), but the effect of systemic administration was not altered by TTX inactivation of NAc or AMG (Figure 3E; one-way ANOVA followed by Bonferroni multiple comparison; P = 0.5376). Hence, after medium-term abstinence, AMPH can reduce tonic VTA activity trough vHPC, AMG, and NAc, but the latter two regions are not required for the effects of systemic re-administration to occur. In this context of short and medium term abstinence from and reintroduction of AMPH, vHPC inactivation was not investigated as vHPC is known to govern VTA via NAc (Grace, 2016), and NAc-inhibition by TTX did not alter effects of readministered AMPH (Figure 3E).

In rats, increased population activity due to abstinence of AMPH for >10 days after repeated exposure was reverted by inactivation of vHPC (Lodge and Grace, 2008). Here, following 11–15 days of abstinence, tonic DA neuron firing was significantly enhanced, and this hyperdopaminergic activity was rescued by TTX inactivation of vHPC (Figure 3F; two-way ANOVA followed by Bonferroni multiple comparison; treatment: P = 0.4451; circuit manipulation: df(1,22) = 26.61, P = 0.0006, interaction: df(1,22) = 29.42, P = 0.0003). Local re-administration of AMPH into NAc, AMG or vHPC reduced DA neuron activity as did systemic application (Figure 3G; one-way ANOVA followed by Bonferroni multiple comparison; P = 0.2383), but inactivation of NAc, AMG, VP, or vHPC by TTX did not interfere with the reduction achieved through systemic administration (Figure 3H; one-way ANOVA followed by Bonferroni multiple comparison; df(4,35) = 6.736, P = 0.0005). Hence, hypo- and hyperdopaminergic states in the VTA due to either ≤4 or >10 days of AMPH abstinence after repeated exposure involve alterations in AMG/NAc and vHPC, respectively. However, for the depression of tonic DA firing by AMPH re-exposure additional brain circuits appear to be recruited.

### 3.3 Activation of mGluR2, but not mGluR3, reverses hypodopaminergia elicited by AMPH short-term abstinence and reduces avoidance behavior

To assess roles of group II mGluRs, the non-selective group II mGluR agonist LY354740, the selective mGluR2 PAM LY487379, and the selective mGluR3 agonist LY2794193 were administered systemically to mice that had experienced repeated AMPH exposure followed by three to 4 days of abstinence. While LY2794193 left VTA activity depressed, as seen with vehicle, respective values were restored towards baseline by LY354740 and LY487379 (Figure 4A; one-way ANOVA followed by Bonferroni multiple comparison; df(3,22) = 22.86, P < 0.0001). Thus, activation of mGluR2, but not mGluR3, rescued hypodopaminergia.

VTA hypodopaminergia due to AMPH abstinence was found to be accompanied by behavioural signatures of anxiety (Rincón-Cortés et al., 2018). Therefore, AMPH-exposed and withdrawn mice received LY487379 and thereafter underwent LDT tasks (see Methods). Controls injected with vehicle exhibited latencies to enter the dark chamber of 161.3 ± 31.61 s and numbers of crosses into the dark chamber of 24.29 ± 3.01 (N = 14; indicated by dashed lines). In AMPH-treated mice tested during short-term abstinence, LY487379 increased the first latency to escape the bright chamber (Figure 4B1; Unpaired t-test; t(28) = 2.509, P = 0.0091) and reduced the total number of trespasses into the dark (Figure 4B2; Unpaired t-test; t(28) = 3.350, P = 0.0012) as compared to vehicle-treated mice. Moreover, activation of mGluR2 reduced mouse navigation (locomotor activity) inside the dark compartment (Figure 4B3; two-way ANOVA followed by Bonferroni multiple comparison; interaction, P = 0.140; treatment, df(1,56) = 5.91, P = 0.0183; compartment, df(1,56) = 6.63, P = 0.0127). Thus, enhancement of tonic DA neuron firing is paralleled by behavioural responses indicative of anti-anxiety actions.

As NAc inactivation reverted hypodopaminergic states in VTA neurons towards baseline (Figure 3C), the two selective mGluR2/3 activators were applied directly into this region. Infusions of LY487379 into NAc completely restored DA neuron activity to baseline, but NAc deposits of LY2794193 had no effect (Figure 4C; one-way ANOVA followed by Bonferroni multiple Comparison Test; df(2,19) = 25.99, P < 0.0001). These results mirror those of systemic administration and highlight NAc as key area for mGluR2 activation to rescue VTA hypodopaminergia associated with short-term abstinence. Given the pre-synaptic localization of mGluR2, we reasoned that a block of glutamate transmission within NAc might counteract effects of LY487379. Accordingly, local deposits of kynurenic acid (Floresco et al., 2001) prevented the actions of systemic LY487379, and VTA activity remained depressed (Figure 4D; one-way ANOVA followed by Bonferroni multiple comparison; df(2,16) = 23.41; P < 0.0001). To reveal whether mGluR2 activation might act via the direct or indirect NAc-VTA pathway, TTX was delivered into VP of mice after three to four days of AMPH abstinence. VP-inactivation prevented effects of systemic LY487379 on VTA DA neuron activity (Figure 4D). Thus, activation of mGluR2 restored tonic DA neuron activity after short-term abstinence via the indirect pathway. However, effects of AMPH re-exposure after short-term abstinence were not affected by either LY354740 or LY487379 (Figure 4E; one-way ANOVA followed by Bonferroni multiple comparison; P = 0.5544).

### 3.4 Activation of mGluR3 in vHPC restores hyperdopaminergic VTA activity elicited by prolonged AMPH abstinence at physiological levels

**FIGURE 5:**
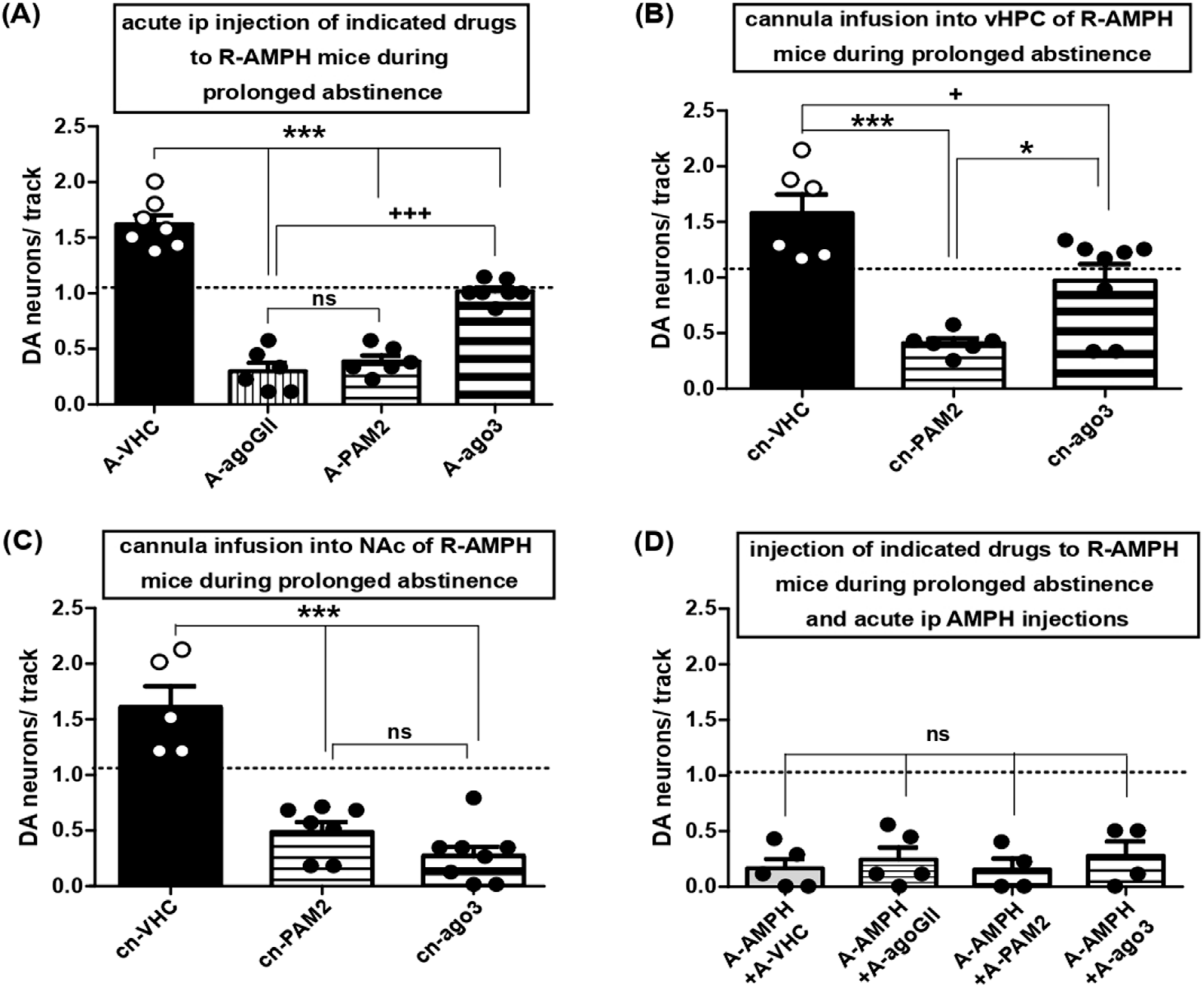
Activation of mGluR3, but not mGluR2, restores hyperdopaminergic VTA activity at baseline levels: role of vHPC and lack of effect on AMPH re-exposure. **(A)** Impact of acute i.p. injections of VHC (A-VHC: N = 7, n = 77), the general group II mGluR agonist LY354740 (3 mg/kg; A-agoGII: N = 6, n = 15), the selective mGluR2 PAM LY487379 (30 mg/kg; A-PAM2: N = 6, n = 19), the selective mGluR3 agonist LY2794193 at 10 mg/kg (A-ago3 (10): N = 7, n = 56), and 5 mg/kg (A-ago3 (5): N = 6, n = 50) on VTA DA neuron activity in mice during prolonged abstinence after repeated AMPH exposure. ***P < 0.001 vs. A-VHC, +++ P < 0.001 vs. A-agoGII ns P > 0.05 vs. A-agoGII; (one-way ANOVA followed by Bonferroni multiple comparison; df (3,25) = 90.97, P < 0.0001). **(B)** Impact of direct infusion of VHC (cn-VHC: N = 6, n = 70), of the selective mGluR2 PAM LY487379 (30 μM; cn-PAM2: N = 6, n = 19), or of the selective mGluR3 agonist LY2794193 (10 μM; cn-ago3: N = 8, n = 63) via acutely implanted cannulas into vHPC of mice during prolonged abstinence after repeated AMPH exposure on VTA DA neuron activity. ***P < 0.0001 vs. cn-VHC, *P < 0.05 vs. cn-VHC; + P < 0.05 vs. cn-PAM2 (one-way ANOVA followed by Bonferroni multiple comparison; df (2,19) = 16.49, P = 0.0001). **(C)** Impact of direct infusion of VHC (cn-VHC: N = 5, n = 55), of the selective mGluR2 PAM LY487379 (30 μM; cn-PAM2: N = 7, n = 24), or of the selective mGluR3 agonist LY2794193 (10 μM; cn-ago3: N = 8, n = 18) via acutely implanted cannulas into NAc of mice during prolonged abstinence after repeated AMPH exposure on VTA DA neuron activity. ***P < 0.0001 vs. cn-VHC; ns P > 0.05 vs. cn-PAM2 (one-way ANOVA followed by Bonferroni’s multiple comparison; df (2,19) = 32.99, P < 0.0001). **(D)** Impact of i.p. injection of VHC (A-VHC: N = 5, n = 6), the general group II mGluR agonist LY354740 (3 mg/kg; A-agoGII: N = 5, n = 11), the selective mGluR2 PAM LY487379 (30 mg/kg; A-PAM2: N = 4, n = 4), or the selective mGluR3 agonist LY2794193 (A-ago3: N = 4, n = 8) in mice during prolonged abstinence from repeated AMPH exposure and injected i.p. with AMPH (2 mg/kg; A-AMPH) on VTA DA neuron activity. ns P > 0.05 vs. A-VHC (one-way ANOVA followed by Bonferroni multiple comparison; P = 0.8158). Dashed lines indicate baseline DA neuron population activity.

Systemic administration of either LY354740 or LY487379 to mice after 11–15 days of abstinence turned average numbers of spontaneously active VTA DA neuron from >1.5 to <0.5 (Figure 5A); LY2794193, however, scaled DA neuron firing down towards baseline levels only (Figure 5A), which pointed towards a reversal of the hyperdopaminergia associated with prolonged AMPH abstinence.

To unveil the sites of drug actions, mGluR activators were applied into vHPC as inactivation of that region was sufficient to reduce hyperdopaminergic VTA activity due to prolonged abstinence from AMPH (Figure 3A; one-way ANOVA followed by Bonferroni multiple comparison; df(3,25) = 90.97, P < 0.0001), which is consistent with previous studies in rats (Lodge and Grace, 2008). Thereby, LY487379 reduced DA neuron activity again to values <0.5, whereas LY2794193 re-established this measure at baseline (Figure 5B; one-way ANOVA followed by Bonferroni multiple comparison; df(2,19) = 16.49, P = 0.0001). For comparison, deposits of LY487379 or LY2794193 into NAc led to average DA neuron counts <0.5 pointing towards VTA hypodopaminergia (Figure 5C; one-way ANOVA followed by Bonferroni’s multiple comparison; df(2,19) = 32.99, P < 0.0001). Moreover, hypodopaminergic states due to AMPH re-administration after long-term abstinence were not affected by group II mGluR activation (Figure 5D; one-way ANOVA followed by Bonferroni multiple comparison; P = 0.8158). Taken together with data concerning AMPH re-exposure after five to six days of abstinence, this indicates that activation of group II mGluRs was not able to interfere with hypodopaminergic VTA activity elicited by AMPH re-exposure.

## 4 Discussion

To shortly summarize the above results, an acute exposure of mice to AMPH reduced VTA DA neuron activity by recruiting the direct NAc VTA pathway. After repeated exposure, VTA DA neuron activity remained reduced for four days and then rose to a hyperdopaminergic state within 15 days. The initial hypoactivity depended on an AMG - NAc - VTA pathway, whereas the hyperactivity relied on vHPC. VTA hypoactivity was recovered towards physiological levels by activation of mGluR2, but not mGluR3; VTA hyperactivity, in contrast, was normalized by selective activation of mGluR3, but not mGluR2. AMPH re-exposure after abstinence turned VTA activity down again, but this suppression could no longer be rescued by mGluR activation. Below, these findings will be discussed point by point.

### 4.1 Reduction of tonic VTA DA neuron activity by acute AMPH administration

A first contact of a mammalian organism with psychostimulants leads to enhanced DA release in VTA output structures and promotes reinforcement and reward-learning (Volkow et al., 2017; Schultz, 2019). However, in accordance with the opponent motivational process theory (Koob and Le Moal, 2008), the hours subsequent to AMPH exposure are characterized by a hypodopaminergic state, which is associated with anhedonia and anxiety as experienced by human patients (Koob and Le Moal, 2008; Lodge and Grace, 2008; Valenti et al., 2021). Here, we confirmed that a reduction of tonic DA neuron firing by AMPH can be observed within half an hour as shown before ((Lodge and Grace, 2008; Valenti et al., 2021) but see (Belujon et al., 2016)). In rats, this decline is maintained by up to 48-h (Belujon et al., 2016).

Even though depression of tonic DA neuron firing has been observed with other psychostimulants, such as cocaine or caffeine (Lodge and Grace, 2008; Valenti et al., 2021), the precise kinetics as well as the sites of AMPH action with respect to VTA DA neuron activity remained to be nailed down. We found that activation of MSN via D1 receptors elicited AMPH-like responses. Moreover, depression of DA neuron firing was elicited by AMPH via multiple VTA afferent regions, these being vHPC, PFC, and AMG, and via NAc. However, only inactivation of NAc, but not of upstream areas like AMG and vHPC, prevented the impact of systemic AMPH on VTA. Hence, AMG, vHPC, and possibly other regions that orchestrate VTA DA neurons do so through the NAc rather than via direct projections to VTA. In addition, acute effects of AMPH were not altered by inactivation of VP thereby favouring a role of a direct NAc-VTA pathway. This is in line with the observation that blockade of D1, but not D2, receptors was able to interfere with AMPH-induced locomotion and conditioned behaviour (O’Neill and Shaw, 1999). Even though the segregation of MSN populations within the NAc-VTA circuit has been questioned (Kupchik et al., 2015), cocaine, but not morphine, was confirmed to impinge on MSNs of the direct pathway to inhibit VTA DA neuron firing in rodents (Edwards et al., 2017). Neurons of the NAc lateral shell, in contrast, were found to disinhibit DA neurons indirectly via VTA GABAergic interneurons (Yang et al., 2018), but it remained unknown whether this pathway might contribute to AMPH-induced variations in population activity as described above. Finally, imaging studies in humans also point towards an imbalance between D1-MSN and D2-MSN as correlate of drug abuse (Kalivas, 2008; Volkow et al., 2013). Taken together, these pieces of evidence favour a role of a direct NAc-VTA connection as central mediator of acute AMPH actions.

### 4.2 Changes in tonic VTA DA neuron activity after repeated AMPH exposure followed by abstinence and effects of group II mGluR activation

Having established key aspects of a first AMPH contact, we turned towards repeated drug exposure followed by abstinence and re-exposure. After repeated AMPH intake, VTA activity remained in a hypodopaminergic state for four days. During the following days, the number of active DA neurons rose in a quasi-linear manner and after intermittent baseline values resulted in a hyperdopaminergic state at days 11–15. While amplified VTA DA neuron firing after repeated AMPH exposure and prolonged abstinence (10–15 days) together with enhanced locomotion has been observed in rats before (Lodge and Grace, 2008), the hypodopaminergia enduring for four days and temporary baseline activity as observed here indicate a slowly ongoing rewiring of neural networks. The ∼ 4-day hypodopaminergia was eliminated by inactivation of NAc, the hyperdopaminergia after prolonged abstinence was rescued by inactivation of vHPC which confirms previous data obtained in rats (Lodge and Grace, 2008). Re-exposure to AMPH after either five to six or 11–15 days of abstinence brought DA neuron activity from baseline and elevated levels (>1.5), respectively, back to values (<0.5) seen after acute exposure and up to four days of abstinence as well. These effects of re-exposure were not affected by inactivation of either NAc or upstream regions, but were also achieved by local injections into such regions. Thus, repeated cycles of AMPH exposure, abstinence, and re-exposure appear to ‘drag’ the DA system in enduring loops of high and low activity. However, while these high-low cycles go on, neuroplastic changes elicited by the intermittent presence of AMPH continue to evolve. Finally, this interferes within the mesolimbic DA system in a way that systemic effects of AMPH re-exposure cannot be recapitulated simply by actions on the NAc-VTA circuit, but rather appear to be dictated by newly established AMPH-sensitive circuits that may impinge on VTA. However, the elucidation of such additional pathways regulating VTA activity was beyond the scope of this investigation.

Group II mGluRs are widely distributed within the limbic circuits and receptor agonists have been known for quite some time to interfere with drug reward and drug seeking (Moussawi and Kalivas, 2010). Here, activation of group II mGluRs was found to re-establish VTA DA neuron activity states at physiological levels after deregulation of the system by repeated AMPH exposure followed by abstinence. This was observed whether the VTA was in a hypo- or hyperdopaminergic state beforehand due to short- and long-term periods of AMPH abstinence, respectively. Nevertheless, selective activation of either mGluR2 or mGluR3 produced opposing results: VTA hypodopaminergia was restored at control levels by activation of mGluR2, whereas hyperdopaminergia was returned towards physiological values via mGluR3. The former effect could be achieved by local delivery of the mGluR2 PAM into NAc, but was abolished by a block of glutamatergic transmission in NAc and inactivation of VP. Thus, the receptors involved were *bona fide* presynaptic mGluR2 within NAc located on nerve terminals that synapse onto MSN of the indirect pathway. The conversion of the hyperdopaminergic state into quasi physiological VTA activity by systemic administration of the mGluR3 agonist was mimicked by local agonist infusion in vHPC, but turned into hypodopaminergia by deposits within NAc. This exaggerated reduction of VTA DA neuron activity argues in favour of additional upstream areas becoming activated after systemic administration, such as AMG and/or PFC, that counteract the local effect within NAc.

Before, activation of group II mGluRs has been found to inhibit cocaine seeking (Peters and Kalivas, 2006), to attenuate intensified AMPH intake due to previous drug exposure (Kim et al., 2005), and to abate the reinstatement of methamphetamine-seeking (Nawata et al., 2024). With respect to isolated effects of receptor subtypes regarding psychostimulant addiction, mGluR2 was found to be key in mediating cocaine addictive behavior (Cannella et al., 2013), whereas activation of mGluR3 and/or inhibition of mGluR2 signaling has been proposed to be beneficial in the treatment of methamphetamine-addiction (Busceti et al., 2021). The present data indicate that during abstinence after repeated AMPH exposure, rewiring of affected brain circuits may proceed and this can lead to a switch in the relative efficiency by which activation of the two group II mGluR subtypes may counteract addictive behavior. Having noted that activation of these receptors can interfere with consequences of short as well as long-term abstinence from AMPH, one also has to admit that effects of subsequent AMPH re-exposure remain untouched by activators of either receptor. This confirms that re-exposure after abstinence most likely recruits alternative neuronal networks that need to be targeted by supplementary strategies.

### 4.3 Behavioral correlates of AMPH abstinence and group II mGluR activation

Anxiety aside of depression is a major symptom of AMPH abstinence in humans (Hall et al., 1996; Conway et al., 2006). In rats, abstinence from either acute (Rincón-Cortés et al., 2018) or chronic (Barr et al., 2011) AMPH exposure leads to anxiety like behaviour and to reduced VTA DA neuron activity, and the latter can be rescued by benzodiazepines as prototypic anxiolytics (Rincón-Cortés et al., 2018). As hypodopaminergia was restored at baseline by the mGluR2 PAM, but not by the mGluR3 agonist, it appeared obvious to test the former for potential effects in anxiety-like behaviour. In the LDT, which reliably detects actions of anxiolytic as well as anxiogenic agents (Shimada et al., 1995), activation of mGluR2 reduced parameters of avoidance behaviour, i.e., the urge to take actions to abate anxiety-like states and also reduced the navigation in the dark compartment. Hence, the rescue of VTA from hypodopaminergic states by an mGluR2 PAM was correlated with anxiolytic drug effects.

In rodents, hyperdopaminergia has been encountered not only due to prolonged abstinence from repeated AMPH (Lodge and Grace, 2008), but also in a developmental rat model of schizophrenia (Lodge and Grace, 2007) and following psychological stress (Valenti et al., 2011b). In schizophrenic patients, psychotic symptoms can be aggravated by a single dose of AMPH (Abi-Dargham et al., 2009), and this disease can be viewed as a state of endogenous sensitization (Vanderschuren and Kalivas, 2000; Weidenauer et al., 2017). In fact, schizophrenic patients display enhanced DA levels in response to AMPH (Weidenauer et al., 2020). Enhanced VTA DA neuron activity is reduced by typical as well as atypical antipsychotics, whereas the opposite is seen when population activity is not exaggerated in a pathological manner (Valenti et al., 2011a). Thus, a reduction of elevated VTA DA neuron population activity can be taken as a correlate of antipsychotic drug actions.

Activation of group II mGluRs has been known as pharmacotherapeutic strategy to improve psychotic symptoms for more than two decades (Maksymetz et al., 2017; Dogra and Conn, 2022). Originally, respective agonists were reported to counteract the behavioural consequences of NMDA receptor blockage (Moghaddam and Adams, 1998). Recently, administration of the group II mGluR agonist pomaglumetad methionil reversed signatures of psychosis in a developmental rat model of schizophrenia (Sonnenschein and Grace, 2020). Meanwhile, several PAMs of mGluR2 were found to share actions of non-selective group II mGluR agonists in rodents that were viewed as correlates of antipsychotic activity (Maksymetz et al., 2017). More recently, activation of mGluR3 has been reported to rescue schizophrenia-like behaviour (Dogra et al., 2024) and to counteract cognitive deficits that were caused by NMDA receptor antagonism through actions on hippocampal pyramidal neurons (Dogra et al., 2021). Moreover, in the rat PCP model of schizophrenia, activation of mGluR3 by the selective agonist LY2794193 was shown to prevent drug-induced hyperlocomotion (Monn et al., 2018). Here, acute administration of LY2794193 during prolonged abstinence from AMPH tuned hyperdopaminergic VTA activity as correlate of psychotic symptoms down to physiological levels when applied directly into vHPC or systemically. A non-selective group II mGluR agonist and a selective mGluR2 PAM, in contrast, forced VTA population activity to hypodopaminergic levels that are known to correlate with signs of anxiety and anhedonia. Hence, these data appear to favor activation of mGluR3 over mGluR2 as strategy to combat positive and negative symptoms of schizophrenia.

### 4.4 Limitations of the study

While it is well established that functions of VTA DA neurons as central components of the mesolimbic DA system are disrupted by recurrent AMPH use (Volkow et al., 2009; Sesack and Grace, 2010), the correlation of the resulting dysfunction, be it hypo- or hyperdopaminergia, with pathomechanisms of psychiatric diseases such as anxiety or psychosis still remains hypothetical. Both diseases are known to rest on a neurodevelopmental background that impinges on the VTA - NAc DA circuitry (Sonnenschein SF. and Grace Aa, 2020; Hanson et al., 2021). It appears somewhat unlikely that repeated AMPH exposure and subsequence abstinence can model a developmental process lasting for weeks, months, or even years. Hence, differential effects of mGluR2 and mGluR3 activation, respectively, on VTA DA neuron function after disorganization by AMPH may allude to separate fields of therapeutic application, but the extrapolation of the present results to the potential treatment of, e.g., anxiety or psychosis must be viewed with precaution.

### 4.5 Conclusion

In summary, the present results reveal a slowly developing switch of VTA population activity from a hypo-to a hyperdopaminergic state during abstinence after repeated AMPH intake. While the former is contingent on a direct NAc-VTA connection, the latter relies on vHPC activity. VTA hypo- and hyperdopaminergia can be restored to physiological levels by activation of mGluR2 and mGluR3, respectively. AMPH re-exposure after abstinence turns VTA activity down, but this latter effect involves alternative circuits and can no longer be rescued by group II mGluR activation.

## Data Availability

The original contributions presented in the study are included in the article/supplementary material, further inquiries can be directed to the corresponding author.
